# Oxidative and Antioxidative Status of Children with Celiac Disease Treated with a Gluten Free-Diet

**DOI:** 10.1155/2018/1324820

**Published:** 2018-05-02

**Authors:** Grażyna Rowicka, Grażyna Czaja-Bulsa, Magdalena Chełchowska, Agnieszka Riahi, Małgorzata Strucińska, Halina Weker, Jadwiga Ambroszkiewicz

**Affiliations:** ^1^Nutrition Department, Institute of Mother and Child, Warsaw, Poland; ^2^Clinic of Pediatrics, Gastrology and Children's Rheumatology, Pomeranian Medical University, Szczecin, Poland; ^3^Department of Screening and Metabolic Diagnostics, Institute of Mother and Child, Warsaw, Poland

## Abstract

**Aims:**

Oxidative stress is a factor involved in the pathogenesis of celiac disease (CD), possibly affecting the course of the disease and celiac-related complications. We assessed the intensity of oxidative processes and the efficiency of antioxidant defense in children with celiac disease*. Methods*. Group I (*n* = 32) consisted of children with CD treated with a gluten-free diet, and group II (*n* = 24) consisted of healthy children on a traditional diet. Antioxidative and oxidative status was assessed by measurement of serum total antioxidant capacity (TAC), total oxidant capacity (TOC), and oxidized low-density lipoprotein (ox-LDL) and on the basis of oxidative stress index (OSI).

**Results:**

There were no significant differences in serum TAC, TOC, ox-LDL, and OSI between children with CD and healthy children. Cluster analysis showed that the group of children with CD is not homogeneous in terms of serum TAC and TOC levels. About 50% of these children had TAC levels < 1.3 mmol/L and TOC levels > 0.35 mmol/L.

**Conclusions:**

Strict adherence to a gluten-free diet by children with CD seems to be important for maintaining oxidative-antioxidant balance. However, further research is needed to identify factors potentially responsible for increased oxidative stress in some children with celiac disease despite adherence to a gluten-free diet.

## 1. Introduction

Celiac disease (CD) is an autoimmune, gluten-sensitive inflammatory disorder of the small intestine, which occurs in people with a genetic predisposition. It is one of the more common genetic diseases, with a prevalence of from 1 : 100 up to 1 : 200 in European and American populations [1]. Although there is no precise data on the prevalence of this disease in the Polish population, it seems that only a small percentage of all cases are detected. Celiac disease is characterized by a complex interaction between genetic and environmental factors. The disease is caused by a persistent intolerance of gluten, which is a storage protein found in grains. Gluten is actually made up of two main groups of proteins: gliadins, also known as prolamins, and glutenins. Celiac disease-related gluten intolerance involves certain fractions of prolamins, gliadin found in wheat, secalin found in rye, and hordein found in barley [2]. Our understanding of biochemical and immunological aspects and the mechanisms involved in the toxicity of these prolamins (there are also many prolamins in cereals, including rice and corn, that do not have toxic properties) is growing. Nevertheless, research is underway to better understand the pathogenesis of the disease. The mucosal damage in celiac patients is considered to be induced by the interplay between innate and adaptive immune responses to ingested gluten. The studies have shown that the gliadin sequence contains regions that play an important role in CD pathogenesis by exerting cytotoxic or immunomodulatory activity. The other regions are responsible for triggering oxidative stress and inducing the release of proinflammatory cytokines [3–9].

Oxidative stress is caused by increased production of reactive oxygen species (ROS), exceeding the capacity of physiological antioxidant systems [10, 11]. Inflammation and oxidative stress due to an increase in reactive oxygen species and a decrease of antioxidant defenses seem to be involved in the molecular mechanisms of celiac disease. It cannot be excluded that they predispose patients with CD to other autoimmune diseases [6, 12–14]. This seems to be particularly true of undiagnosed or diagnosed but untreated/inappropriately treated disease [15, 16].

Efficient antioxidant mechanisms, both enzymatic and nonenzymatic, defend the body against free radical damage. Antioxidant enzymes include, for example, superoxide dismutase (SOD), catalase (CAT), glutathione peroxidase (GPx), glutathione reductase (GR), glutathione S-transferase (GST), and glucose-6-phosphate dehydrogenase (G6PDH). Nonenzymatic mechanisms include, among others, glutathione (GSH), vitamin C (ascorbic acid), vitamin E (*α*-tocopherol), *β*-carotene, vitamin A (retinol), flavonoids, and binding proteins—transferrin, ceruloplasmin, and albumin [16]. Some nonenzymatic antioxidants are essential diet components and are found in many food products.

Total antioxidant capacity (TAC) is an expression of a complete ability to neutralize free oxygen radicals that initiate oxidative damage. TAC includes, among others, such factors as uric acid (32–65%), thiol groups of proteins (10–50%), ascorbic acid (6–24%), vitamins A and E (5–10%), and albumin-bound bilirubin (7%). Although uric acid and thiols represent the greatest percentage contribution to TAC, their antioxidant capacity is lower compared with vitamins, which are considered highly efficient nonenzymatic antioxidants. Markers that determine the intensity of the oxidation processes include, inter alia, TOC (total oxidant capacity), measuring the total oxidant capacity in the serum, and ox-LDL (oxidized low-density lipoprotein), indicating the intensity of lipid peroxidation [17].

From a clinical point of view, CD is characterized by a clinical heterogeneity that ranges from asymptomatic to severely symptomatic and by increased morbidity and mortality. Strict lifetime adherence to a gluten-free diet is currently the only available, effective, and safe therapy for celiac disease [18, 19].

The question arises whether the use of a strict gluten-free diet in children with CD is sufficient for maintaining a serum oxidative/antioxidant balance in these children.

The aim of the study was to assess the intensity of oxidative processes and the efficiency of antioxidant defense mechanisms in children with celiac disease treated with a gluten-free diet.

## 2. Materials and Methods

The study was carried out according to the principles of the Declaration of Helsinki and approved by the Ethics Committee of the Institute of Mother and Child in Warsaw, Poland. Informed consent was obtained from the parents of the study participants and from children aged over 16 years old. The study was conducted as part of statutory task IMiD number OPK 510-25-48.

### 2.1. Subjects

The study covered 56 children, including 27 girls (48%) and 29 boys (52%) aged between 7 and 18 years, attending the Gastroenterology Outpatient Clinic of the Institute of Mother and Child in Warsaw and the Gastroenterology Outpatient Clinic of Children's Hospital “Zdroje” in Szczecin (SPS ZOZ “Zdroje”). Children with celiac disease diagnosed in accordance with the European Society for Pediatric Gastroenterology Hepatology and Nutrition (ESPGHAN) were included in group I (*n* = 32). These were children on a strict gluten-free diet, as evidenced by the absence of serum IgA and IgG anti-transglutaminase (tTG) antibodies in at least the last year [18]. The control group II included 24 healthy children, whose serological screening detected to be negative and who had no history of any chronic disease.

Exclusion criteria were acute and chronic inflammation, consumption of dietary supplements containing substances with antioxidant activity, and chronic comorbidities that could increase oxidative stress in children with celiac disease.

### 2.2. Anthropometric Measurements

The children's nutritional status was assessed on the basis of weight and height. Weight (kg) and height (m) were used to calculate body mass index (BMI) as body weight (kg) divided by height squared (m^2^). BMI values were compared with BMI norms for age and sex according to OLAF criteria, thus obtaining a BMI z-score, which is a normalized relative weight indicator independent of age and sex [20].

### 2.3. Blood Sampling and Biochemical Analysis

For biochemical measurements, venous blood (3.0 mL) was taken in the morning hours from fasting patients. Blood was collected in the usual manner, but the full blood count sample was collected into anticoagulated tubes with sodium heparin. In order to obtain plasma, the blood was centrifuged at 2500 ×g at 4°C for 10 minutes. Plasma and serum samples were frozen (−70°C) until measurements of IgA and IgG anti-human tTG antibodies (IgA tTG ab, IgG tTG ab) and concentrations of biochemical parameters (TOC, TAC—max 4 weeks, ox-LDL, vitamin E, ferritin, and uric acid—max 6 months) were performed. The tTG antibody serum levels were assessed using a commercial FluoroEnzymeImmuno Assay kit (Phadia, Sweden), and a value below 7 U/mL was considered negative [21]. Serum total oxidative capacity (TOC) and total antioxidative capacity (TAC) were evaluated by colorimetric/photometric assay using commercially available kits (LDN Labor Diagnostika Nord GmbH & Co. KG, Germany). Determinations of TOC and TAC are based on the reaction of peroxides with peroxidase followed by a color reaction of the chromogenic substrate tetramethylbenzidine. The analytical sensitivity of TOC was 0.06 mmol/L, and the intra- and interassay coefficients of variation (CV) were 4.90% and 7.33%, respectively. The sensitivity of TAC was 0.08 mmol/L, and the intra- and interassay CV were 5.00% and 6.92%, respectively [22].

According to the manufacturer's reference data (manual of LDN Labor Diagnostika Nord GmbH & Co. KG, Germany), the expected value of TOC was <0.35 mmol/L, while TAC indicating sufficient antioxidative capacity was >1.3 mmol/L and indicating borderline antioxidant capacity was between 1.0 and 1.3 mmol/L. Also, in the study of Drabko and Kowalczyk [23], healthy children had a mean value of total antioxidant status at the level of 1.3 mmol/L.

Oxidative stress index (OSI) was defined as the percentage ratio of TOC levels to TAC levels.

Oxidized-LDL (ox-LDL) levels were determined by enzyme-linked immunosorbent assay (ELISA) (Immundiagnostik AG, Bensheim, Germany). The intra- and interassay coefficients of variability were found to be less than 5.7% and 9.0%, respectively. The detection limit was 4.13 ng/mL.

Plasma concentrations of vitamins A (retinol) and E (*α*-tocopherol) were measured by high-performance liquid chromatography (HPLC, KNAUER, Germany) using a methodology based on the procedure of Zaman et al. [24].

Serum levels of C-reactive protein (CRP), ferritin, and uric acid as well as total cholesterol (TC), HDL cholesterol (HDL-C), LDL cholesterol (LDL-C), and triglyceride (TG) concentrations were determined using standard methods on the Integra Cobas 400 plus analyzer (Roche Diagnostics, Switzerland).

### 2.4. Statistical Analysis

The appropriate difference significance tests, such as Student's *t*-test for variables with normal distribution and homogeneity of variance as well as the Mann–Whitney *U* test for variables with nonnormal distribution, were used to assess the differences between children with CD and the controls in terms of the analyzed anthropometric and biochemical variables.

Data are presented as mean values and standard deviations for variables with normal distribution or medians and interquartile ranges for variables with nonnormal distribution. The Shapiro-Wilk test was used to test the normality of variable distributions.

Pearson's correlation coefficients (Pearson's *r*) were calculated for oxidative and antioxidant status indicators and other biochemical parameters as well as selected anthropometric and clinical variables. Cluster analysis (CA) was performed with the *k*-means method to identify groups of children differing in their oxidative and antioxidant status. Two quantitative variables, TOC and TAC, as well as a qualitative variable for group membership (CD/healthy children) were used for CA. *p* < 0.05 was considered statistically significant. Statistica 12 PL was used for statistical analysis.

## 3. Results

The characteristics of the studied children are presented in Table 1.

The percentage of girls and boys was similar in both groups of children, with 50%/50% in group I and 45.8%/44.2% in group II. Children with celiac disease were on a gluten-free diet for an average of 7 years.

Children in both groups did not differ significantly in terms of age, body weight, or height. Although BMI and BMI z-score were significantly lower in children with CD, the value of BMI z-score pointed to an appropriate nutritional status of children from both groups (BMI z-score <−1;+1>).

As shown in Table 2, there were no significant differences in serum TAC, TOC, ox-LDL, and OSI between patients with CD and healthy children. Vitamin A and ferritin levels were significantly higher in patients with CD compared with healthy children (*p* < 0.05). There were no significant differences in mean serum vitamin E and uric acid levels between the two groups.

Correlations between serum concentrations of oxidative-antioxidative status markers and clinical/biochemical parameters in children with CD are presented in Table 3.

A positive correlation was found between ferritin and TOC levels (*p* < 0.05) as well as HDL cholesterol and ox-LDL levels (*p* < 0.01) in the group of children with celiac disease. Additionally, a negative correlation between vitamin A and ox-LDL levels (*p* < 0.01) as well as HDL and OSI (*p* < 0.05) was shown in this group.

**Figure 1:**
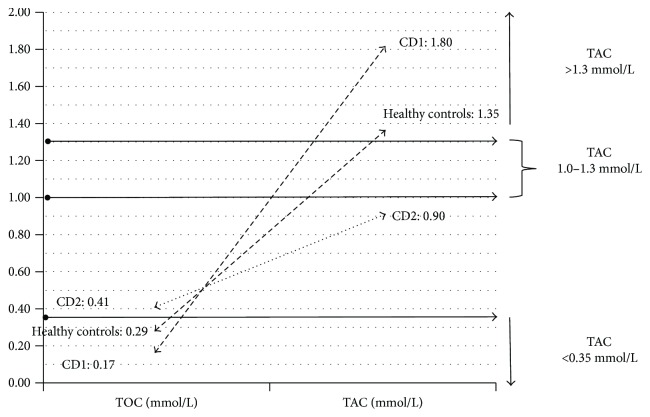
Clusters of children differing in terms of TAC and TOC levels. CD1: children with celiac disease with concentrations of TAC > 1.3 mmol/L and TOC < 0.35 mmol/L. CD2: children with celiac disease with concentrations of TAC < 1.3 mmol/L and TOC > 0.35 mmol/L. Healthy controls with concentrations of TAC > 1.3 mmol/L and TOC < 0.35 mmol/L.

Based on cluster analysis performed with the *k*-means method, three groups (clusters) of children with varying TOC and TAC levels including two groups of children with celiac disease were identified: CD1 (*n* = 17/56) with mean TAC levels of 1.80 mmol/L and mean TOC levels of 0.167 mmol/L; CD2 (*n* = 15/56) with TAC/TOC levels of 0.89 mmol/L and 0.41 mmol/L, respectively; and the third group consists of healthy children (*n* = 24/56) with mean TAC levels of 1.34 mmol/L and mean TOC levels of 0.29 mmol/L (Figure 1).

Cluster analysis showed that the group of children with celiac disease is not homogeneous in terms of serum TAC and TOC levels, with about 50% of these patients (CD2 cluster, *n* = 15/32) with TAC levels considered insufficient for proper antioxidant defense (<1.3 mmol/L) and TOC levels indicating increased oxidative processes (TOC > 0.35 mmol/L) (Figure 1).

**Figure 2:**
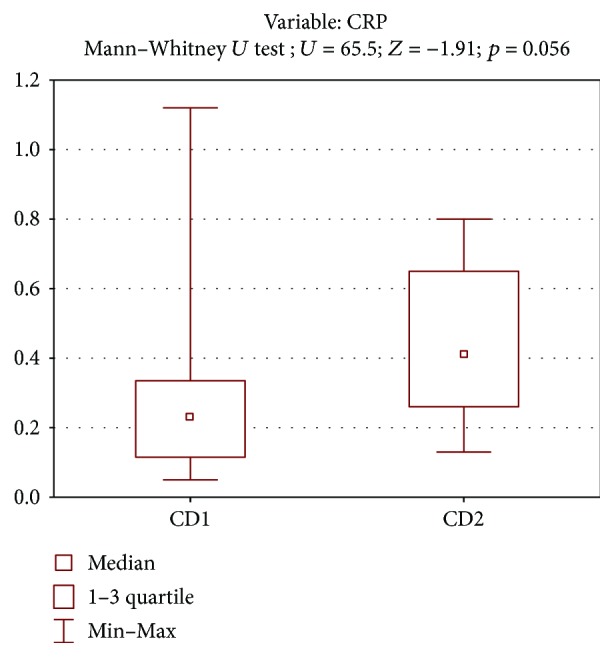
Differences in CRP level between CD1 and CD2 children.

Differences in CRP level between CD1 and CD2 children are presented in Figure 2.

Children with celiac disease (CD1) with TOC and TAC levels within the range of expected values had lower CRP levels (median 0.23; interquartile range 0.12–0.34) compared with children with celiac disease (CD2) with serum TOC and TAC levels not corresponding to the expected values (median 0.41; interquartile range 0.26–0.65) (Figure 2).

## 4. Discussion

Consumption of gluten by patients with celiac disease induces the overproduction of reactive oxygen species, triggering a cascade of reactions that cause oxidative stress both at the small intestinal mucosa and the whole-body level. Oxidative stress is responsible for free radical damage of important cellular structures, thus adversely modifying their functions [25–28]. A number of studies have been conducted to assess selected markers of antioxidant and oxidative processes in children with celiac disease [6, 15, 29–31]. Our study was conducted as a part of the discussion on whether a gluten-free diet is sufficient for maintaining a serum oxidative/antioxidant balance in children with celiac disease. This may seem doubtful considering the fact that despite strict adherence to the diet (for over 12 months), some celiac patients still experience persistent or recurrent symptoms of refractory celiac disease (RCD) [32]. In the available literature, there are few studies using markers such as TAC, TOC, ox-LDL, and OSI to assess oxidative/antioxidant imbalance in children with celiac disease. Our study demonstrated no differences in the intensity of oxidative/antioxidant processes assessed based on the above parameters between children with celiac disease treated with a gluten-free diet and healthy children, although the levels of parameters such as ferritin and vitamin A included in TAC were significantly higher in the celiac group. This could suggest that a gluten-free diet is sufficient for maintaining oxidative/antioxidant balance. Nevertheless, a subgroup of children experiencing oxidative stress, despite strict diet adherence, was identified among patients with CD.

The literature data indicates that the antioxidant potential assessed based on various markers both in intestinal mucosa and bodily fluids (blood, urine) is significantly lower in newly diagnosed celiac patients compared with healthy individuals, while the intensity of oxidation processes is higher [6, 15, 29–31]. The results suggest that gliadin disturbs the pro−/antioxidant balance through the overproduction of ROS not only in the small intestinal mucosa of affected persons [33]. An interesting observation was presented by Stojiljković et al. [15], who demonstrated that the severity of the mucosal lesion in celiac patients significantly correlated with the evaluated markers of radical damage. Thus, a positive correlation was found between the severity of histological lesions in the intestinal mucosa and SOD activity as well as LOOH (lipid peroxides) levels, and the activities of CAT, GPx, and GR and the concentration of GSH inversely correlated with the degree of the mucosal lesion. It seems that the lower antioxidant potential in individuals with untreated celiac disease may be due to, among others, an increased demand for antioxidants necessary to compensate for the elevated production of ROS, and thus prevent their adverse effects. Intestinal mucosal damage caused by reactive oxygen species, leading to secondarily impaired absorption of antioxidant nutrients, may also play an important role [30].

A gluten-free diet is usually associated with clinical improvement, the disappearance of serological markers specific for the disease, and regeneration of the small intestinal mucosa in patients with celiac disease. This should be accompanied by significant improvement in the oxidative/antioxidant balance. The beneficial effects of a gluten-free diet on antioxidant status were demonstrated by Ferretti et al. [34]. The authors showed that although serum TAC levels were indeed lower in patients with celiac disease who used a gluten-free diet compared with the controls, these levels were still significantly higher compared with newly diagnosed patients who were not on a gluten-free diet. In contrast to Ferretti et al., our study showed no significant differences in mean TAC levels between celiac patients on a gluten-free diet and healthy children. It cannot be excluded that in addition to a gluten-free diet, diet duration is also important for maintaining oxidative/antioxidant balance; however, our study showed no significant correlation in this regard. A diet consisting of natural antioxidants also seems to play an important role. It was documented that several dietary components exert anti-inflammatory and antioxidant roles and have a protective effect on the intestinal epithelium [35–37]. In our study, serum vitamin A levels in children with celiac disease were significantly higher compared with those of the controls, while *α*-tocopherol levels did not differ between the groups. Szaflarska-Poplawska et al. [13] showed no significant differences in serum vitamin A and E levels between children with celiac disease treated with a gluten-free diet and healthy children; however, the levels of these antioxidants were significantly lower in untreated versus treated patients with CD. Vitamin E, as a scavenger of free oxygen radicals, protects polyunsaturated fatty acids (PUFA), a major structural component of the cellular membranes, from peroxidation [38]. Synergistic interactions between vitamin E and vitamin A against lipid peroxidation were documented [39]. In our study, dietary intake of these vitamins in children with celiac disease could have affected the lack of significant differences not only in TAC and TOC levels and OSI values but also in ox-LDL levels between children with celiac disease and healthy children. This may be indicated by the inverse correlation between vitamin A and ox-LDL levels found in the group of children with celiac disease. Furthermore, we also observed a reverse correlation between HDL cholesterol, demonstrating anti-inflammatory and antioxidative activities, with OSI in this patient population. At the same time however, a positive correlation was found between HDL cholesterol and ox-LDL, which would suggest HDL's effect on oxidative stress contrary to the expected one. Nevertheless, OSI seems to better define oxidative stress severity.

Sayar et al. [29] demonstrated that IMA (ischemia-modified albumin stress marker), TOS (total oxidant status), and AOPP (advanced oxidation protein products) levels were significantly lower after just 30 months of a gluten-free diet, while TAC and SH sulfhydryl levels were significantly higher. Similarly in the study by Ertekin et al. [40], serum nitric oxide levels were found to be higher in children with CD than in the control group, and these levels were found to regress after implementing a gluten-free diet. These findings indicated that oxidative stress regressed after the introduction of a gluten-free diet in celiac patients. However, we showed in our study that despite strict adherence to the diet, about 50% of children with celiac disease (CD2) had relatively low TAC levels and high TOC levels, which would indicate oxidative stress. In the same group of children, CRP levels were higher compared with children with celiac disease (CD1) with relatively high TAC and low TOC levels, yet still within the range of reference standards. These results seem to confirm the findings of other authors. Stojiljković et al. [15] point to the fact that oxidative stress may also occur in children with CD who are on a gluten-free diet, as evidenced by differences in the activity of antioxidant enzymes and glutathione (GSH) observed in this group. Szaflarska-Poplawska et al. [13] also observed significant differences in the levels of markers for free radical DNA damage (8-oxodG in DNA isolated from leukocytes and in urine samples) in children with celiac disease compared with healthy children; however, they found no such differences between these parameters in children with treated and untreated celiac disease. Similarly, the study by Odetti et al. [30] indicates that a redox imbalance persists in celiac disease even when asymptomatic. The authors found an increase of markers of oxidative damage of lipids (thiobarbituric acid-reactive substances and lipid hydroperoxides) and proteins (carbonyl groups) in the blood serum of patients with celiac disease compared with healthy individuals. According to the authors, the redox imbalance is probably caused by an absorption deficiency, even if slight. Thus, they suggest that dietary supplementation with antioxidant molecules may offer some benefits. These results suggest that a gluten-free diet is able to only partially improve the functionality of the intestinal mucosa, which leads to the necessity to conduct further studies on this topic.

Our study has some limitations. Firstly, the study included relatively small groups of children, although comparable to those investigated by other authors. Additionally, the group of children with celiac disease was homogeneous, as all children strictly adhered to a gluten-free diet as evidenced by the absence of serum antibodies against tissue transglutaminase. Secondly, data on the content of antioxidants, such as vitamins A, C, and E, or LC-PUFA (long-chain polyunsaturated fatty acid) in the children's diets were missing. However, considering the increased vitamin A levels among children with celiac disease and similar levels of vitamin E in both groups, it can be assumed that the diets of these children were balanced in this regard.

*To conclude*, strict adherence to a gluten-free diet by children with celiac disease seems to be an important condition for maintaining oxidative-antioxidant balance, as evidenced by a lack of differences in TAC, TOC, ox-LDL, and OSI levels between children treated due to celiac disease and the controls. However, further research is needed to identify factors potentially responsible for increased oxidative stress in some children with celiac disease despite adherence to a gluten-free diet.

## Figures and Tables

**Table 1:** Characteristics of the studied children.

Variables	Children with CD (*n* = 32)	Healthy controls (*n* = 24)	*p* value
Age (years)^+^	13.8 (10.4–16.5)	12.4 (8.4–14.4)	0.089
Treatment with gluten-free diet (years)^++^	7.6 (3.3)	—	—
Weight (kg)^+^	43.2 (28.3–52.5)	42.2 (36.9–59.8)	0.144
Height (cm)^++^	153.8 (19.1)	153.5 (17.2)	0.952
BMI (kg/m^2^)^++^	17.3 (3.4)	19.6 (2.4)	0.006^∗^
BMI z-score^+^	−0.7 (−1.0 to −0.3)	0.4 (−0.4–1.0)	0.0001^∗^

^+^Data are presented as median value and interquartile ranges (1Q–3Q). ^++^Data are presented as mean value and standard deviation (SD). ^∗^Statistically significant differences (*p* < 0.05). BMI: body mass index; BMI z-score: a normalized relative weight indicator independent of age and sex.

**Table 2:** Biochemical parameters in children with CD and healthy controls.

Variables	Children with CD (*n* = 32)	Healthy controls (*n* = 24)	*p* value
TOC (mmol/L)^+^	0.23 (0.13–0.35)	0.27 (0.16–0.36)	0.608
TAC (mmol/L)^++^	1.38 (0.60)	1.35 (0.42)	0.815
Ox-LDL (ng/mL)^+^	125.4 (68.6–242.0)	147.4 (56.5–446.8)	0.796
OSI^+^	0.16 (0.07–0.36)	0.20 (0.14–0.27)	0.535
CRP (mg/L)^+^	0.29 (0.15–0.50)	0.40 (0.14–0.90)	0.355
Ferritin (ng/mL)^+^	81.0 (33.1–120.0)	43.4 (26.3–60.1)	0.027^∗^
Uric acid (mg/dL)^+^	4.4 (4.0–5.4)	4.4 (4.1–4.9)	0.868
Vitamin E (*μ*mol/L)^+^	17.8 (15.3–19.7)	18.7 (14.7–20.9)	0.952
Vitamin A (*μ*mol/L)^++^	2.0 (0.42)	1.6 (0.56)	0.026^∗^
Cholesterol total (mg/dL)^++^	167.7 (24.6)	166.9 (24.5)	0.903
Cholesterol HDL (mg/dL)^++^	56.7 (21.8)	59.8 (15.1)	0.567
Cholesterol LDL (mg/dL)^+^	97.0 (83.0–126.0)	95.5 (81.5–117.0)	0.601
Triglycerides (mg/dL)^+^	68.5 (48.0–104.0)	61.0 (48.0–100.0)	0.781

^+^Data are presented as median value and interquartile ranges (1Q–3Q). ^++^Data are presented as mean value and standard deviation (SD). ^∗^Statistically significant differences (*p* < 0.05). TOC: total oxidant capacity; TAC: total antioxidant capacity; ox-LDL: oxidized low-density lipoprotein; OSI: oxidative stress index; CRP: C-reactive protein.

**Table 3:** Correlations between serum concentrations of oxidative-antioxidative status markers and clinical/biochemical parameters in children with CD (*n* = 32).

Variables	TOC		TAC		OSI		ox-LDL	
	*r*	*p*	*r*	*p*	*r*	*p*	*r*	*p*
Age	−0.14	0.448	0.11	0.549	−0.07	0.711	−0.05	0.813
Treatment with gluten-free diet	0.38	0.055	−0.18	0.387	0.21	0.313	0.33	0.096
BMI z-score	−0.02	0.928	−0.11	0.546	−0.08	0.664	−0.05	0.810
CRP	−0.20	0.293	−0.20	0.294	−0.06	0.751	0.02	0.928
Ferritin	0.43	0.014^∗^	0.10	0.573	0.15	0.422	0.17	0.386
Uric acid	−0.17	0.415	0.05	0.825	−0.09	0.667	−0.13	0.551
Vitamin E	−0.27	0.190	0.33	0.097	−0.35	0.080	−0.07	0.749
Vitamin A	0.06	0.775	−0.14	0.492	0.15	0.461	−0.55	0.003^∗^
Cholesterol total	0.26	0.169	0.17	0.380	0.15	0.421	0.12	0.567
Cholesterol HDL	−0.30	0.109	0.18	0.350	−0.38	0.038^∗^	0.49	0.009^∗^
Cholesterol LDL	0.32	0.089	0.02	0.920	0.31	0.100	−0.18	0.364
Triglycerides	−0.03	0.872	−0.04	0.846	−0.02	0.904	−0.22	0.266

*r*: Pearson's correlation coefficient, ^∗^*p* < 0.05; TOC: total oxidant capacity; TAC: total antioxidant capacity; ox-LDL: oxidized low-density lipoprotein; OSI: oxidative stress index; CRP: C-reactive protein; BMI z-score: normalized relative weight indicator independent of age and sex.
